# Supplementary material for: Flatten the curve: Empirical evidence on how non-pharmaceutical interventions substituted pharmaceutical treatments during COVID-19 pandemic
Source: PLoS One. 2021 Oct 11;16(10):e0258379. doi: 10.1371/journal.pone.0258379 (PMC8504736; doi:10.1371/journal.pone.0258379)
Supplement: Attachment — Submitted filename: Response to Reviewers.docx [file pone.0258379.s001.docx]

**Response to Reviewer Comments**

Sept. 13, 2021

**Manuscript ID:**

Dear Editor and Reviewer,

In accordance with the comments of the reviewer in this round of revision, we have revised the above manuscript, “Flatten the Curve: Empirical Evidence on How Non-Pharmaceutical Interventions Substituted Pharmaceutical Treatments during COVID-19 Pandemic”. We have addressed all the comments from the reviewers and provided our responses as follows. Please note that the revisions made in the revised manuscript are marked in yellow color according to the reviewer’s comments.

Thank you again for reviewing our paper. We will look forward to hearing from you.

Sincerely,

Weiyu Luo

Wei Guo

Songhua Hu

Mofeng Yang

Xinyuan Hu

Chenfeng Xiong

**Reviewer #1:**

1) There are some other regression models, why choose the GAM model in the analysis?

**Response:** The GAM model enables researchers to capture non-linearities between the response variable and multiple predictors by smooth functions. In this research, we are most interested in modeling the mutual non-linear effect of pharmaceutical and non-pharmaceutical variables on the response variable. Therefore, we choose the GAM model in our analysis.

2) Some variables in Tables 3 and 5 are insignificant.

**Response:** We thank the reviewer for pointing this out. Several variables like “Weekday or Weekend”, “Sex” and “Federal Emergency tag” are insignificant. However, we decide to reserve these variables as covariates to control weekly and social-demographic variation and pattern.

3) Compared to the existing literature, what are the novel contributions from this study?

**Response:** Thanks for this comment. As far as we checked the existing literature, our research is among the first to quantify the mutual effect of non-pharmaceutical factors and pharmaceutical factors on pandemic. This research may provide empirical suggestions to help government make trade-offs between non-pharmaceutical factors and pharmaceutical factors in the future, therefore help optimize the policy making to benefit the whole society. *Please see Section 1, Introduction:*

*“The main contributions of this research can be summarized as follows. First, this research leverages the aggregated data from large amounts of location-based service data which is more informative and accurate than assumptions on disease transmission rate or reproduction rate (Ray et al., 2020). Second, most of the previous researchers solely focused on NPTs or PTs, and no one has studied the substitution effect of NPT and PT. This research is also among the first to quantify the relationship between NPTs and PTs between NPTs and PTs, and to visualize their substitutional impact. This is innovative and timely research that will provide practical information to the medical system. It also provides a good prediction on New Death Rate, New Case Rate, and New Mild Case Rate. Third, this study contributes to broader general interests. Utilizing the daily updated web portal (Maryland Transportation Institute, 2020) on the pre-trained models mentioned above, we can deliver days- or weeks-ahead predictions to the public.”*

4) This study considers many different explanatory variables. One approach to overcome the model uncertainty is the Bayesian model averaging model and this approach can improve the model prediction. The authors are suggested to review and discuss this approach in the study. For example, see: Application of the bayesian model averaging in analyzing freeway traffic incident clearance time for emergency management. Journal of advanced transportation, 2021. Housing appraisal under model uncertainty: Bayesian model averaging method. Advanced Engineering Journal, 1(1), 26-34.

**Response:** We thank the reviewers for pointing this out. We reviewed several literatures and *added some discussion in Section 6, Conclusion and Discussion:*

*“Despite this research is mainly focusing on finding the substitution effect, one next step on making better predictions is also significant. Several previous research suggest methods to overcome the model uncertainty and make better predictions. For instance, we might explore applying different types of models and aggregate them by Bayesian Model Averaging methodology (Erdoğdu et al., 2021; Zou et al., 2021).”*

For the next step, we will focus on the improvement of the model predictions by applying the Bayesian model averaging.

**Reviewer 2:**

1) Data source: The reviewer suggests the authors provide a more in-depth discussion on their dataset. Although the reviewer acknowledges that the dataset from MTI has been used widely, more description of the data process method and limitation of this dataset should be discussed. For example, does the MTI dataset include location obtained from cell phone trajectory?

**Response:** We have added some sentences describing the data source in *Section 2, Data Source:*

*“This platform incorporated over 150 million anonymous individuals monthly active mobile devices to develop the dataset of person movements for the U.S. These data are collected from individual devices including iOS and Android OS. The risk of re-identification is reduced by applying privacy-preserving techniques, such as aggregating to census block group level or county level. More technical details can be found in this paper (Hu et al., 2021) and the project report (Zhang et al., 2020b).”*

2) Table 1: Please consider providing sources of all variables.

**Response:** We agree and have updated the information as a *new “Source” column in Section 3, Variable Explanation, Table 1. The updated table is as below:*

| **Abbreviation** | **Explanation** | **Type** | **Source** |
| --- | --- | --- | --- |
| *Dependent Variables* | |  |  |
| New Death Rate | 7-day moving average of Daily New Covid-19 death number per 1 million people of each state. | - | From JHU repository (https://github.com/CSSEGISandData/COVID-19/tree/master/csse_covid_19_data). |
| New Case Rate | 7-day moving average of Daily New Covid-19 confirmed case number per 1 thousand people of each state. | - | Calculated by MTI based on JHU repository. |
| New Mild Case Rate | 7-day moving average of Daily New Covid-19 mild case number per 1 thousand people of each state. Calculated by (new confirmed case number – new death number of 7 days later)/population*1000 | - | - |
| *Independent Variables* | |  |  |
| Weekday | 1 for Monday, 7 for Sunday and 2 to 6 are in between. | - | - |
| TI | Time Index. Days offset from 01/01/2020 as day 0. | - | - |
| age60 | 60 years old population percentage | Sociodemographic | Census Bureau |
| Inc | Median income | Sociodemographic | Census Bureau |
| Afr | African Americans population percentage | Sociodemographic | Census Bureau |
| Hisp | Hispanic Americans population percentage | Sociodemographic | Census Bureau |
| Male | Male percentage | Sociodemographic | Census Bureau |
| PD | People per square mile (Population Density) | Sociodemographic | Census Bureau |
| Hot | Number of points of interests for crowd gathering per 1000 people | Sociodemographic | Calculated by MTI |
| HB | Number of staffed hospital beds per 1000 people | Sociodemographic | ESRI: US Hospital Beds Dashboard |
| FE | Federal emergency tag for each day. 0 for not declaring, 1 for declaring. | - | - |
| Appr | Gubernatorial approval ratings | - | https://ballotpedia.org/Gubernatorial_approval_ratings |
| DTest24 | Number of COVID-19 daily tests per 1000 people. The data is lagged for **24 days** and applied to a 7-day moving average. | Pharmaceutical Treatment | https://covidtracking.com/data/api |
| Ctrip24 | Number of trips per 100 people that cross county borders with origin and destination in the same state. The data is lagged for **24 days** and applied to a 7-day moving average. | Non-Pharmaceutical Treatment | Calculated by MTI |
| Strip24 | Number of trips that cross state borders per 100 people. The data is lagged for **24 days** and applied to a 7-day moving average. | Non-Pharmaceutical Treatment | Calculated by MTI |
| ICU24 | Percentage of ICU units occupied with COVID-19 patients. The data is lagged for **24 days** and applied to a 7-day moving average. | Pharmaceutical Treatment | ESRI: US Hospital Beds Dashboard |
| DTest11 | Number of COVID-19 daily tests per 1000 people. The data is lagged for **11 days** and applied to a 7-day moving average. | Pharmaceutical Treatment | https://covidtracking.com/data/api |
| Ctrip11 | Number of trips per 100 people that cross county borders with origin and destination in the same state. The data is lagged for **11 days** and applied to a 7-day moving average. | Non-Pharmaceutical Treatment | Calculated by MTI |
| Strip11 | Number of trips that cross state borders per 100 people. The data is lagged for **11 days** and applied to a 7-day moving average. | Non-Pharmaceutical Treatment | Calculated by MTI |
| ICU11 | Percentage of ICU units occupied with COVID-19 patients. The data is lagged for **11 days** and applied to a 7-day moving average. | Pharmaceutical Treatment | ESRI: US Hospital Beds Dashboard |

3) Model: The reviewer challenges the validity of using the state as a unit of analysis. First, people’s COVID-19 relevant behavior (e.g., perceived perception, travel behavior, wearing masks, and so on) might vary considerably within each state. For example, in many Midwest states, we have observed huge differences between urban and rural residents. Also, given the fact that people’s response to COVID-19 is highly politicized in the U.S., using the state as the unit of analysis cannot capture this important aspect successfully.

**Response:** We thank the reviewer for pointing this out. We agree with the reviewer and have upgraded our model to a county-level model. We have used each county in the US as a unit of analysis. This approach increased the number of observations from 5550 to 301536. All the corresponding tables, figures and sentences were updated.

4) Model: Moreover, the reviewer understands the potential issue of inter-state trips of the D.C. metro area. This inter-state trip is also important for other parts of the U.S. (e.g., Chicago MSA: IL-IN-WI, Cincinnati MSA: OH-KY-IN). Please provide more details about how the authors address this issue. Also, provide more details about how Figure 1 is created (e.g., data source). Figure 1 has not been addressed in the manuscript.

**Response:** Thank you so much for pointing this out. As mentioned above, we have upgraded our model to a county-level model, the D.C. metro area is kept in the training dataset, so this issue no longer exists now.

5) Page 5: The manuscript assumes that “the dependent variable follows a Gamma distribution.” Could the authors please provide scientific references or justification for this assumption?

**Response:** Thank you for pointing this out. We reviewed and cited several literatures regarding the gamma distribution assumption of virus reproduction number. *These three papers are listed below:*

*Lloyd-Smith, J. O., Schreiber, S. J., Kopp, P. E., & Getz, W. M. (2005). Superspreading and the effect of individual variation on disease emergence. Nature, 438(7066), 355–359.*

*Pasetto, D., Lemaitre, J. C., Bertuzzo, E., Gatto, M., & Rinaldo, A. (2021). Range of reproduction number estimates for COVID-19 spread. Biochemical and Biophysical Research Communications, 538, 253–258.*

*Zhao, H., Merchant, N. N., McNulty, A., Radcliff, T. A., Cote, M. J., Fischer, R. S. B., Sang, H., & Ory, M. G. (2021). COVID-19: Short term prediction model using daily incidence data. PLoS ONE, 16(4 April), 1–14.*

The sentences are modified *in Section 4.1,* *GAM and data preprocessing:*

*“Several research assumed that the virus reproduction numbers are gamma distributed in transmission modeling (Lloyd-Smith et al., 2005; Pasetto et al., 2021; Zhao et al., 2021), here we also use log link function assuming the dependent variable followings a Gamma distribution applying the log link function on the left-hand side of the formula.”*

6) Page 6: Could the authors please provide more justification for choosing 21 and 14 days for different models?

**Response:** Thank you for pointing this out. In the final model, we changed the lag days to 24 for the death model and 11 for the case model. Referring to the results from “Association between mobility patterns and COVID-19 transmission in the USA: a mathematical modelling study” (Badr et al., 2020), an optimal lag of days of 11 is calculated by optimizing the correlation between the pre-defined “mobility ratio” and “COVID-19 growth rate ratio”. Referring to the results from “Case-Fatality Risk Estimates for COVID-19 Calculated by Using a Lag Time for Fatality” (Wilson et al., 2020), the median time delay is 13 days from illness onset to death. The number “13 days” can also be supported by “Intensive care management of coronavirus disease 2019 (COVID-19): challenges and recommendations” (Phua et al., 2020) which said “the median time from symptom onset to severe hypoxaemia and ICU admission is approximately 7-12 days”. Therefore, we use 11 + 13 = 24 days as the lag number for the death model. *The sentences were added in Section 4.3, Model Selection:*

*“Referring to the previous research (Badr et al., 2020), an optimal lag of days of 11 is calculated by optimizing the correlation between the pre-defined “mobility ratio” and “COVID-19 growth rate ratio”, therefore we chose 11 days for the New Case Rate and New Mild Case Rate models. Referring to the previous research (Wilson et al., 2020), the median time delay is 13 days from illness onset to death. The number “13 days” can also be supported by another previous research (Phua et al., 2020) which said “the median time from symptom onset to severe hypoxaemia and ICU admission is approximately 7-12 days”. Therefore, we chose used 11+13=241 days as the lag number for the New Death Rate model.”*

7) Table 1: Some technical terms in the abbreviated form need a brief explanation (e.g., e.d.f.? s(F.E., bs=”re”), and so on).

**Response:** We agree the reviewer’s comment on this. We have added several explanations for “e.d.f”, “bs=’re’”, “R-sq.”, “deviance explained” *in Section 4.3, Model Selection:*

*“In the lower half, the smooth terms with parameter “bs=’re’” are set to produce a random coefficient for each level of the factor. The abbreviation “e.d.f” is short for “effective degrees of freedom”, which reflects the non-linearity of each smooth term. A larger “e.d.f” represents more wiggliness of the smooth term. The adjusted R-sq. is calculated by the formula as follows:*

$${Adjusted R}^{2}=1-\frac{\sum_{i} \left( y_{i}-f_{i} \right)^{2}/(n-k)}{\sum_{i} \left( y_{i}-\bar{y} \right)^{2}/(n-1)}$$

*The percentage of deviance explained (Hastie & Tibshirani, 1990) in this paper is calculated as below:*

*% Deviance Explained=(Deviance_Null-Deviance_Residual)/(Deviance_Null )×100%*

*Where deviance is defined as:*

$$D\left( y,\hat{\mu} \right)=2(\log\left( p\left( y | \hat{\theta_{s}} \right) \right)-\log\left( p\left( y | \hat{\theta_{0}} \right) \right))$$

- *Null deviance:* $\theta_{0}$ *refers to the null model (i.e., intercept-only model).*
- *Residual deviance:* $\theta_{0}$ *refers to the trained model.*
- $y$ *represents the outcome.*
- $\hat{\mu}$ *represents the estimate of the model.*
- $\hat{\theta_{s}}$ *and* $\hat{\theta_{0}}$ *are the parameters of the fitted saturated and proposed models, respectively. A saturated model has as many parameters as it has training points, that is, 𝑝=n.*
- $p(y|\theta)$ *is the likelihood of data given the model.*

8) Page 8: The manuscript states that “The R-sq. of the New Death Rate model is remarkably higher than the New Case Rate […], reflecting the data for New Death Rate is more trustworthy than for New Case Rate.” The reviewer challenges this argument because the model fit indices do tell less thing about data quality. In other words, data trustworthiness should not be judged by the model fit indices.

**Response:** This observation is correct; we thank the reviewer for pointing this out. In the original manuscript, the author tried to explain the reason why the New Death Rate model performed better but made a less rigorous inference. We have revised this sentence *in Section 5.1, Inferential Analysis into:*

*“The “R-sq.” and “deviance explained” of the New Death Rate model are higher than the New Case Rate and New Mild Case Rate models, reflecting a better explanation of variances and a better improvement from null model to the fitted model for New Death Rate than for New Case Rate from the predictors.”*

9) Page 12: The results illustrated in Figure 4 are interesting. However, the reviewer wonders why the authors particularly focused on this time of period to check their predictability of the model? Have the authors considered applying the model to the most recent timeframe (e.g., September 2020 – January 2021) to check whether the model also works well?

**Response:** We thank the reviewer for pointing this out. In general, people’s travel behavior and medical system are influenced the most at the initial stage of a novel epidemic. During that period, unknown attributes of a novel disease make it difficult for people and government to make decisions. This is the reason we wanted to focus our model specifically on the initial stage of the COVID-19 outbreak.

10) After reading the entire manuscript, the reviewer hopes to ask one important question: How did (or will plan to) the authors address the bi-directional relationship between human mobility and the spread of the COVID-19 virus. This manuscript only considers a one-way directional influence of human mobility on the COVID-19 (e.g., new cases). However, in reality, this would be more of a complex relationship that affects each other. Also, people’s changing risk perception (over time) might significantly affect their behaviors and eventually affect the number of new COVID-19 cases. Could the authors please discuss this issue?

**Response:** We thank the reviewer for this comment. For the first question, one approach is to build another model to check the opposite one-way relationship between the two variables (Xiong et al., 2020). In the next step, we also plan to try another approach. This approach is to introduce SEM (Structural Equation Modeling) methodology to address the bi-directional relationship between human mobility and the spread of the COVID-19 virus in a single model. To check whether it’s a one-way or two-way directional influence, we plan to set both variables as endogenous variables and train multiple baseline models to compare. We also reviewed one paper regarding the SEM application (Mueller & Hancock, 2011; Zong et al., 2019). For the second question, we agree the reviewer that people’s risk perception should be taken into consideration in the model. People’s risk perception over time may be influenced by many aspects, for example, change of the government order, stay-at-home fatigue, new vaccine availability, etc. To capture the perception changing, we may try to utilize the social media data such as Facebook, Twitter, Instagram and so on as the input of our model. Several studies have utilized social media data to extract people’s perception on COVID-19 related incidents like travelling (Yu et al., 2020) , lockdown (Li et al., 2021) and the risk mitigation strategies (Malecki et al., 2021). We added a paragraph addressing these two questions *in Section 6, Conclusion and Discussion:*

*“This research mainly discussed a one-way relationship between human mobility and the spread of the COVID-19 virus. However, the opposite direction or the bi-directional relationship also exist between the two variables. Previous research explored the opposite direction on these two variables (Xiong et al., 2020a). For the next step, the authors also plan to apply SEM (Structural Equation Modeling) methodology (Mueller & Hancock, 2011; Zong et al., 2019) to explore further on the bi-directional relationship. In addition, people’s risk perception over time might significantly affect their behaviors and affect the spread of the virus. In this case, we plan to utilize social media data such as Facebook, Twitter, Instagram and so on as input to our model to capture people’s changing in the risk perception. Several studies have utilized social media data to extract people’s perception on COVID-19 related incidents like travelling (Yu et al., 2020), lockdown (Li et al., 2021) and risk mitigation strategies (Malecki et al., 2021).”*

References:

Badr, H. S., Du, H., Marshall, M., Dong, E., Squire, M. M., & Gardner, L. M. (2020). Association between mobility patterns and COVID-19 transmission in the USA: a mathematical modelling study. *The Lancet Infectious Diseases*, *0*(0), 1–8. https://doi.org/10.1016/S1473-3099(20)30553-3

Li, L., Ma, Z., Lee, H., & Lee, S. (2021). Can social media data be used to evaluate the risk of human interactions during the COVID-19 pandemic? *International Journal of Disaster Risk Reduction*, *56*(February), 102142. https://doi.org/10.1016/j.ijdrr.2021.102142

Malecki, K. M. C., Keating, J. A., & Safdar, N. (2021). Crisis Communication and Public Perception of COVID-19 Risk in the Era of Social Media. *Clinical Infectious Diseases*, *72*(4), 697–702. https://doi.org/10.1093/cid/ciaa758

Mueller, R. O., & Hancock, G. R. (2011). Best Practices in Structural Equation Modeling. *Best Practices in Quantitative Methods*, 488–508. https://doi.org/10.4135/9781412995627.D38

Phua, J., Weng, L., Ling, L., Egi, M., Lim, C. M., Divatia, J. V., Shrestha, B. R., Arabi, Y. M., Ng, J., Gomersall, C. D., Nishimura, M., Koh, Y., & Du, B. (2020). Intensive care management of coronavirus disease 2019 (COVID-19): challenges and recommendations. *The Lancet Respiratory Medicine*, *8*(5), 506–517. https://doi.org/10.1016/S2213-2600(20)30161-2

Wilson, N., Kvalsvig, A., Barnard, L. T., & Baker, M. G. (2020). Case-fatality risk estimates for COVID-19 calculated by using a lag time for fatality. *Emerging Infectious Diseases*, *26*(6), 1339–1341. https://doi.org/10.3201/EID2606.200320

Xiong, C., Hu, S., Yang, M., Luo, W., & Zhang, L. (2020). Mobile device data reveal the dynamics in a positive relationship between human mobility and COVID-19 infections. *Proceedings of the National Academy of Sciences of the United States of America*, *117*(44), 27087–27089. https://doi.org/10.1073/pnas.2010836117

Yu, M., Li, Z., Yu, Z., He, J., & Zhou, J. (2020). *Current Issues in Tourism Communication related health crisis on social media: a case of COVID-19 outbreak.* https://doi.org/10.1080/13683500.2020.1752632

Zong, F., Yu, P., Tang, J., & Sun, X. (2019). Understanding parking decisions with structural equation modeling. *Physica A: Statistical Mechanics and Its Applications*, *523*, 408–417. https://doi.org/10.1016/j.physa.2019.02.038
